# Oncogenic RAS in Cancers from the DNA Replication Stress and Senescence Perspective

**DOI:** 10.3390/cancers16233993

**Published:** 2024-11-28

**Authors:** Hervé Técher, Samira Kemiha, Xieraili Aobuli, Arun Mouli Kolinjivadi

**Affiliations:** 1 Université Côte d’Azur, Institute for Research on Cancer and Aging of Nice—IRCAN, CNRS, INSERM, 06100 Nice, France; 2 Lee Moffitt Cancer Center, 12902 Magnolia Drive, Tampa, FL 33612, USA; 3 Cancer Science Institute of Singapore, National University of Singapore, Singapore 117599, Singapore

**Keywords:** cancers, RAS, genomic instability, senescence, DNA replication, DNA damage response

## Abstract

Rat Sarcoma (RAS)-driven cancers have been one of the main foci in the field of cancer science for over four decades. Despite significant improvement in understanding the biology of RAS oncogene, the method to target RAS-mutated cancers is still unclear. In recent years, the role for RAS beyond its hyperproliferation has been extensively documented. In this review, we systematically address and dwell on the details of the mechanisms of RAS oncogene-mediated alteration in the DNA replication and DNA damage response (DDR) pathways, focusing on lung cancers. We further extend this molecular connection towards cytosolic DNA accumulation, innate immune activation and senescence in RAS-addicted cancers. At the end, we briefly speculate on the potential strategies for targeting RAS mutated lung cancers, considering various approaches targeting DNA replication, DNA repair and the cGAS-STING pro-inflammatory pathway. These new lines of therapy, especially when used in combinations, may enhance treatment efficacy and overcome the challenges associated with these mutations.

## Simple Summary

Oncogenes are proteins promoting cancer formation. Cancers caused by mutations in the RAS oncogene have been a major focus of cancer research for decades. Despite better understanding of how the RAS oncogene works, finding an effective way to treat RAS-driven cancers remains a challenge. This review explores how RAS affects DNA replication and genome stability. It also describes how RAS can lead to inflammatory responses and senescence. Finally, we discuss how these molecular insights have led to potential new lines of treatments to target RAS-mutated cancers.

## 1. Introduction

**Figure 1:**
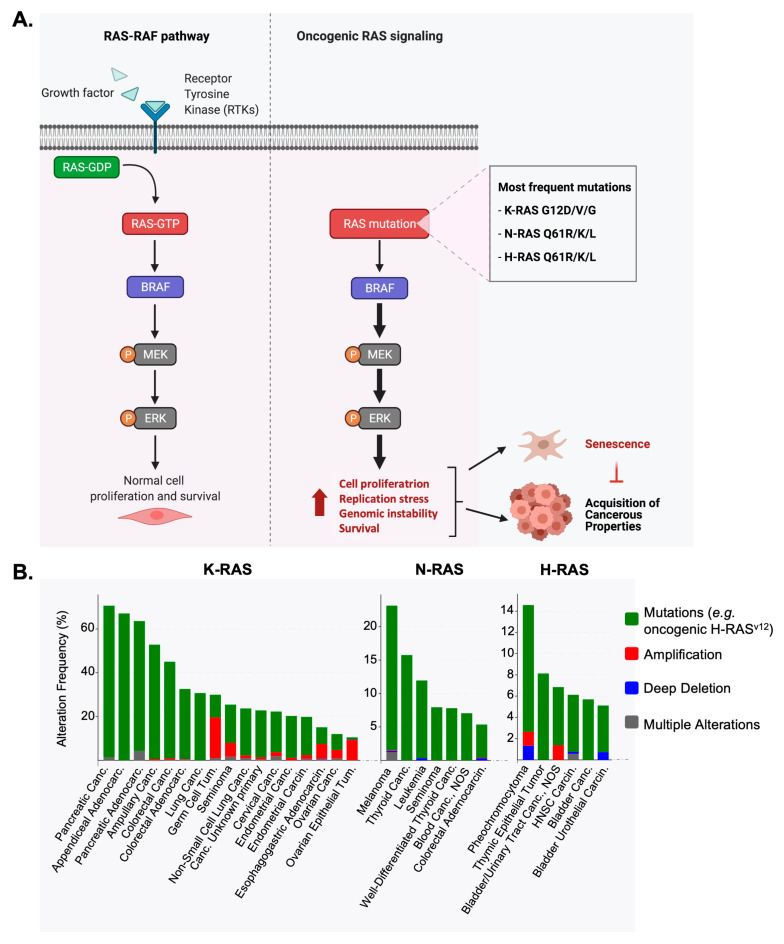
RAS signaling and mutations in cancers. (**A**) Schematic of RAS signaling in normal tissue (left panel) and cancers (right panel). RAS proteins are small GTPases that are used as signaling messengers to convey growth factor sensing by Receptor Tyrosine Kinase (RTK, e.g., EGFR epidermal growth factor receptor). Active RAS binds to GTP and activates downstream signaling kinases. Among the main ones is the BRAF-MEK-ERK pathway, which controls transcriptional activation of target genes involved in cell proliferation and survival. In cancers, RAS is very frequently mutated (see (**B**)); as such, it acquires auto-activation properties which unables it to hydrolyze GTP and as such maintains RAS in an active form. As such, RAS signaling is aberrantly ‘switched-ON’ in the absence of biological cues, namely growth factors. RAS mutations are leading to hyperproliferation, alterations in DNA replication dynamics, genomic instability and increased survival. Two main outcomes are then possible either in whatsoever normal cells: aberrant RAS activity leads to senescence (oncogene-induced senescence, OIS), or if secondary mutations are acquired that permit cells to escape senescence, they can further transform and take the direction to tumor growth. This figure has been created with Biorender.com (**B**). Frequencies of genomic alterations of K-RAS, N-RAS and H-RAS as retrieved from the cBioportal for Cancer Genomics database (www.cbioportal.org) (accessed on 3 April 2021) [8,9]. Data were retrieved from 185 non-redundant pan-cancers studies (TCGA and non-TCGA) with non-overlapping samples. A threshold of at least 50 cases/patients per study was used. Histograms are shown per each cancer type with studies with >10% or >5% of alteration frequency for K-RAS and H-RAS/N-RAS, respectively.

Cancers can be described in broad terms as the acquisition over time of genetic alterations that lead to uncontrolled proliferation and the incapacity to eliminate these aberrant sub-populations of cells. These concepts are best explained in the classical ‘Hallmarks of cancer’ which when first postulated in the year 2000 brought to light ‘six essential alterations in cell physiology that collectively dictate malignant growth’ [1]. Among those six, four essential properties that define cancers are, for instance: ‘self-sufficiency in growth signals, insensitivity to growth-inhibitory (antigrowth) signals, evasion of programmed cell death (apoptosis), limitless replicative potential’ [1]. The acquisition of these hallmarks during tumor formation depends primarily on the activation of oncogenes, such as MYC, ERK, ERBB2 (HER2-neu) or RAS (Rat Sarcoma). RASs, being the focus of our review, are small GTPases and are prototypal oncogenes. In normal cellular physiology, RAS signaling is an essential transducer of growth factor signaling (Figure 1) [2]. Once mutated, oncogenic RAS may fuel several of these hallmarks of cancer.

Alongside oncogenes, a second class of cancer genes are tumor suppressors (TSs), which serve as a protection against the acquisition of these cancerous hallmarks. Archetypal TS is the DNA damage response (DDR) gene TP53, coding for the ‘guardian of the genome’ p53 protein [3]. Strikingly, TP53 is altered in approximately half of human cancers. The ‘hallmarks of cancers’ were later updated to include two very important features of cancers that have proven a decade later to be the state of the art in cancer research, as explained through our manuscript [4]. On one side, there is genomic instability and DNA replication defects, and on the other side, inflammation and immune response, which have been and still are on the central stages of progress in cancer research and in the development of new lines of therapy [4]. These two key features of cancers are both important for the comprehension of cancer formation and to find new treatments, notably to overcome chemo-resistance. Furthermore, it is now well understood that inflammation and genome instability are connected and impact both cancer formation and response to treatments [5,6,7].

In this review, we discuss in detail the mechanisms of how RAS oncogene fuels genome instability and senescence, which are involved in the early stages of cancer initiation and later during the progression. We focus primarily on the recent findings made in lung cancers, highlighting the results obtained in non-small cell lung cancers (NSCLCs). Furthermore, we dwell more on the details of the effect of RAS in altering DNA replication mechanisms and its molecular connections with inflammatory response and senescence. Finally, we discuss the possible combinations and/or sequential treatments that can be used to target cancers at different levels to improve current treatment modalities.

## 2. RAS in Normal Physiology and Cancers

### 2.1. RAS Family and Its Mutations in Cancers

The RAS family of small GTPases are archetypal oncogenes. In normal cellular physiology, RAS signaling is an essential transducer of growth factor signaling (Figure 1A) [2]. RAS signaling activates the MEK-ERK pathway to modulate the transcription of genes involved in cell proliferation. RAS signaling can also activate the PI3K (phosphoinositide-3 kinases) kinases. RAS mutations mainly occur on the codons 12, 13 and 61. These mutations decrease their GTPase activity, and as such, oncogenic RAS remains bound to GTP in an active form. Many forms of oncogenic RAS thus activate survival and proliferation pathways independently of the presence of growth signals (Figure 1A). Of note, some forms of oncogenic RAS, notably K-RAS G12C, can still be stimulated and further activated by growth factors [2]. Aside from point mutations, RAS genes can be amplified but barely deleted (Figure 1B). RAS exists in three isoforms K-RAS, N-RAS and H-RAS [10]. K-RAS mutations are observed in ~60 to ~40% of pancreatic and colorectal cancers, respectively. N-RAS (~20%) and H-RAS (~14%) are prevalent in melanoma and in pheochromocytomas, respectively (Figure 1B). We focus on non-small cell lung cancers (NSCLCs) for which approximately 20% of patients show a K-RAS activating mutation (e.g., K-RAS G12D or K-RAS G12C) (Figure 1B). NSCLCs are epithelial lung cancers other than small-cell lung carcinoma (SCLC). These types of cancers are insensitive to chemotherapies and thus new therapeutic strategies are needed. K-RAS mutations account for 32% of lung adenocarcinoma [11], a subtype of NSCLCs.

### 2.2. Alterations of the Broad RAS Signaling and Mutational Signature in Lung Cancers

It is important to note here that in addition to RAS genes, other members of the broad RAS signaling pathway can be mutated and behave similarly to increase oncogenic RAS signaling. It is the case with the EGFR (Epithelial Growth Factor Receptor), BRAF, ERK-MAP kinases, ERBB2, and so on and so forth. For instance, the broad RAS signaling pathway is mutated in 74% of lung adenocarcinomas in The Cancer Genome Atlas (TCGA) [12]. In addition to RAS mutations, identifying patients with RAS signaling alterations through monitoring the transcriptional signatures of RAS activity might be crucial. A novel transcriptional signature, RAS84, has been developed to identify cancers that are driven by RAS signaling by non-KRAS mutations [13]. This signature shows that ~80% of lung adenocarcinomas show activation of RAS signaling pathway independently of K-RAS mutation. Transcriptional signature RAS84 is mainly clonal, showing that oncogenic RAS activity is an early driver, at least in NSCLCs (lung adenocarcinomas) [13]. Previously described transcriptional signatures of oncogenic RAS activity, such as the ‘K-RAS addiction’ signature [14,15,16], have been outperformed by this novel RAS84 signature.

Analysis of mutational signatures in cancer samples has been instrumental for learning the history of mutations and define possible mechanisms involved in mutation burden [17]. Different lung cancers, including NSCLCs, show a significant increase in the mutational signatures 1B, 2, 4 and 5 [18,19]. Signature 1B is probably due to elevated rates of 5-methyl-cytosine deamination (C>T). Signature 2 is linked to increased activity of the APOBEC (apolipoprotein B mRNA editing enzyme catalytic polypeptides) family of cytidine deaminase that converts cytidine to uracil. APOBEC mutations may be coupled to base excision repair (NER) or DNA replication. Both signatures 1B and 2 are found in many types of cancers and are not specific to lung cancers. Mutational signature 4 is associated with cancers significantly linked to tobacco smoking. As such, signature 4 may be a ‘genomic scar’ of DNA damages caused by carcinogens found in tobacco smoke, such as polycyclic hydrocarbons, which are removed by transcription-coupled NER. This mutational signature shows a high frequency of C>A mutations on transcribed strands that is consistent with the formation of DNA adducts on guanine. Signature 5 is of unknown origin. Sometimes in lung cancers, and other types of cancers, these events of mutation are clustered into small foci of local mutations, known as kataegis [18]. Therefore, RAS mutations, to some extent, are likely to be generated by carcinogens contained in tobacco smoke or other sources of pollutants. Remarkably, oncogenic KRAS driver mutations are found in around 50% of healthy lung samples [20]. This suggests that pre-existing oncogenic mutation in healthy tissue may be selected for further transformation by secondary events, such as exposure to pollutants, and in this case micro-particles were associated with lung cancer formation [20].

As oncogenic RAS signaling impacts many aspects of cellular biology, it is difficult to define a specific function that RAS alters in cancers. It has been shown that RAS promotes cell proliferation [21,22,23], blocks apoptosis [11], changes the metabolism to sustain hyperproliferation [24] and increases inflammation [25,26]. Since RAS has been proposed to be an early driver during cancer development, the study of normal cells with activated RAS oncogenes has been instrumental for comprehending the early steps of cellular transformation.

## 3. Oncogenic RAS Signaling, Hyperproliferation and DNA Replication Stress

### 3.1. What Are the Early Events Driven by Oncogenic RAS in Normal Cells?

The role of oncogenes, including RAS, in understanding cancer formation has been extensively studied in normal human fibroblasts. In normal human cells, oncogenic RAS leads to oncogene-induced senescence (OIS) [27]. In brief, mutant RAS overexpression leads to a first stage of hyperproliferation that is accompanied by alterations in DNA replication dynamics with associated DNA damage. These DNA lesions are then sensed by the DDR sensors leading to cellular senescence. Senescence is a specific form of proliferation arrest, and as such represents a way to put a brake on abnormal cellular transformation driven by oncogenic activities [27,28]. Classical markers of senescence are senescence-associated-β-galactosidase (SA-β-gal) staining, persistence of DNA damage (γH2AX, 53BP1 foci), markers of cell-cycle inhibition with p16 or p21 positivity, senescence-associated heterochromatin foci (SAHF, i.e., large patterns of condensation of the chromatin) and the secretion of pro-inflammatory cytokines and chemokines, known as senescence-associated secretory phenotype (SASP) [29]. It has further been proposed that DDR is a barrier during cancer development [30]. Inactivation of DDR genes, which carry TS functions, by secondary mutations would enable transforming cells to cross this barrier and acquire cancerous properties.

Two main causes of OIS have been described as follows: (i) perturbations of DNA replication and associated ATM-p53 (ataxia-telangiectasia mutated)-mediated DDR activation [31,32] and (ii) activation of the cytosolic DNA sensor cGAS (cyclic GMP-AMP Synthase) [33,34,35]. These two molecular mechanisms may not be exclusive and may be connected at different levels, as explained in the next sections.

### 3.2. Replication Stress Is a Major Source of Genome Instability in RAS-Transformed Cells

Oncogenes induce replication defects, known as replication stress (RS), which is associated with the accumulation of DNA damages and chromosomal aberrations [31,32,36]. Oncogene induced-RS is mainly detected as slow traveling forks using single molecule DNA fiber methods [37,38]. It is notably the case in fibroblasts overexpressing (OE) the oncogenic mutant H-RASG12V (i.e., H-Ras^v12^) [32,39,40]. Fork slowing, used as a readout of RAS-induced RS, has been consistently observed by independent groups [32,40,41,42,43]. Whether other known cancer driver RAS mutations do alter DNA replication has long remained unknown. Fork progression defects were recently reported in both K-RAS G13D and K-RAS G12V mutants [44], suggesting that RS is a general consequence of oncogenic mutations in the RAS family. Alteration of fork progression is a common feature of oncogenic transformation by MYC, or Cyclin E and other oncogenes [40,41,45,46,47]. Oncogenic stress leads to pausing/stalling of elongating forks (i.e., fork asymmetry) [39,43].

Most of these replication defects are accompanied with canonical markers of DNA lesions and DDR signaling (e.g., ATR/ATM signaling, γ-H2AX, Rad51 foci, 53BP1 foci). Consistently mitotic aberrations (e.g., anaphase bridges or lagging chromosomes) and subsequent micronuclei formation increase upon oncogenic stress [39,43,45,48]. Genomic instability is thus recurrent upon oncogenic stress, suggesting that perturbation of DNA replication is one potential source of chromosomal rearrangements and mutations that can promote further cellular transformation. As a confirmation that RS leads to genomic instability in RAS-transformed cells, the mapping of chromosomal breaks identified specific fragile sites, partly overlapping with known common fragile sites (CFSs) that are hard-to-replicate regions of the genome [49]. CFS breakages are usually induced by inhibitors of replication fork progression [50].

### 3.3. What Is the Source of the Replication Stress in RAS-Transformed Cells?

**Figure 2:**
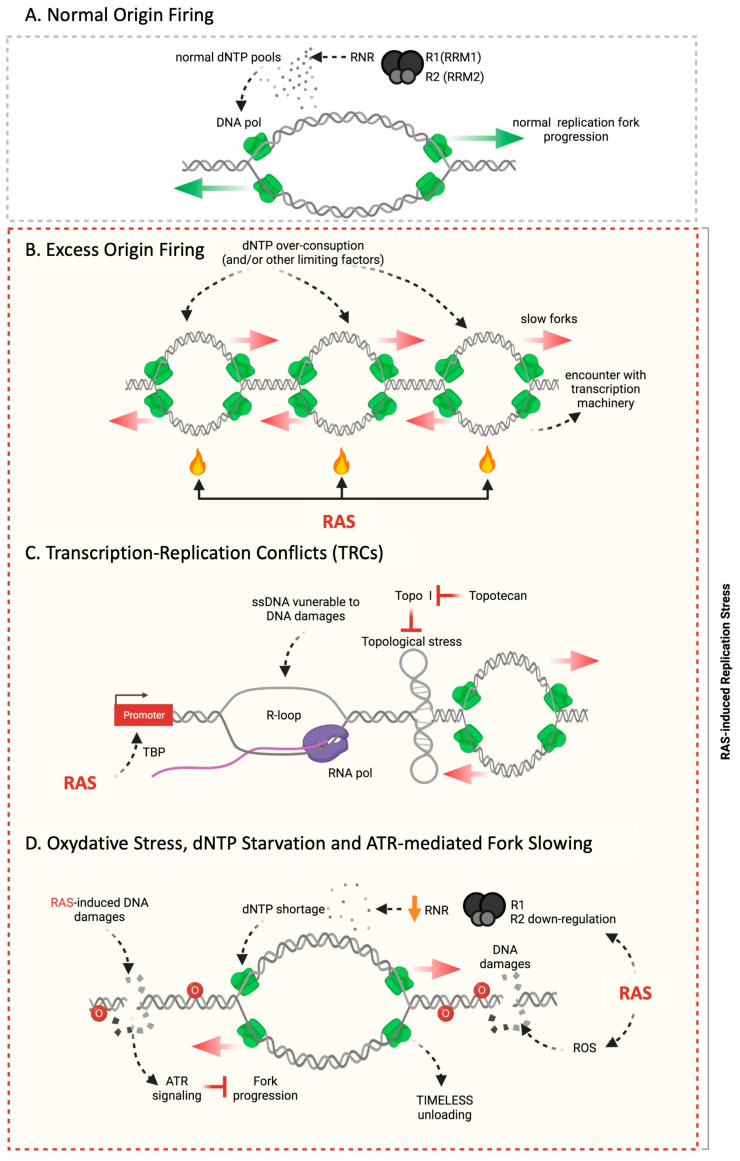
The possible molecular mechanisms of oncogene-induced replication stress. Oncogenic stress, notably induced by H-RAS^v12^ leads to DNA replication defects. (**A**) This panel depicts the activation of origins of replication in normal cells. The replisome (depicted in green), principally made of replicative helicases and DNA polymerases (DNA pol), opens up the DNA double-helix enabling two replication forks to progress in opposite directions. In normal S-phase, the DNA template is undamaged; dNTP pools are available in adequate quantities, which allow the replication forks to progress normally (normal fork progression is depicted by green arrows). (**B**–**D**) Here, we illustrate the different molecular mechanisms that may alter origin firing and impede fork progression upon oncogenic stress. Red arrows show alteration of fork progression. This figure has been created with Biorender.com. See details in the text.

In our view, there are five non-exclusive mechanisms that explain the manifestation of RS, and thus of chromosomal instability, in RAS-transformed cells (see Figure 2).

#### 3.3.1. Hyperproliferation, Excess Origin Firing and Re-Replication

Di Micco and colleagues have shown that the early hyperproliferative phase of RAS-transformed cells is accompanied by increased DNA replication origin firing or initiation [32] (Figure 2A,B). An excess of origins has an impact on the consumption of limiting factors. As such, too many initiation events will eventually deplete the pool of dNTPs, slowing down the progression of DNA replication forks. Excess origin firing and uncontrolled activity of origins may lead to the re-firing of regions of the genome that have already been replicated [32], a phenomenon known as re-replication of chromosomal DNA.

#### 3.3.2. Transcription and Replication Conflicts (TRCs)

Replication fork slowing in RAS overexpressing fibroblasts can be abrogated by RNase H1 overexpression or inhibition of transcription [39]. These results suggest that transcription–replication conflicts (TRCs) are the main cause of fork impediment in RAS-transformed cells (Figure 2C). RNase H1 removes R-loops by degrading RNA at the level of RNA: DNA hybrids. R-loops are RNA: DNA hybrids that form during transcription, displacing a ssDNA strand that forms a small loop [51,52,53]. R-loops are structures that may form preferentially at gene transcription start sites and transcription termination sites, the later location being more prone for the encounter in the head-to-head orientation (head-on) in between the replication and transcription machineries [54,55,56]. RAS activity has a huge impact on transcriptional programs which make the occurrence of R-loops and TRCs more likely. Consistently, R-loop levels increase in H-RAS^v12^ overexpressing cells [39].

#### 3.3.3. Downregulation of dNTP Synthesis

RAS overexpression leads to a decrease in the expression of the small regulatory subunit of ribonucleotide reductase (RRM2, RNR2), a key enzyme in dNTP metabolism (Figure 2D). Mechanistically, it has been shown that the transcription repressor E2F7 binds to RRM2 promoter in RAS overexpressing cells [57]. It has been reported that addition of nucleotide precursors or overexpression of the ribonucleotide reductase to RAS-OE fibroblasts abrogates both fork progression defects and OIS [57]. Consistently, shortage of dNTPs has been reported as the main cause of RS and DNA damage with other oncogenic proteins, namely in either HPV-16 E6/E7 or Cyclin E OE-cells [46]. Considering these findings, it has been proposed that the origin of the RS induced by activated oncogenes stem from metabolic alterations resulting in dNTP shortage [46,57]. Increased rate of origin firing in this case will exacerbate the consumption of the limited pool of available precursors.

#### 3.3.4. Oxidative Stress

The expression of oncogenic RAS has been shown to increase intracellular levels of ROS (reactive oxygen species) (Figure 2D) [58]. Limiting oxygen exposure or supplementing RAS-transforming cells with antioxidants (e.g., NAC) suppressed senescence [59]. These ROS are natural byproducts of cellular metabolism. RAS activity alters cellular metabolism to sustain hyperproliferation and may at the same time increase ROS levels. ROS are very reactive chemicals that damage proteins, DNA molecules and nucleotide precursors. ROS, generated by RAC1 and NOX4, may mainly act as DNA damaging agents in the hyperproliferative stage as proposed by D’Adda di Fagagna and co-workers [59]. How ROS impact fork progression is not well understood yet. One possible mechanism involves peroxiredoxin 2 (PRDX2). PRDX2 forms a replisome-associated ROS sensor, which binds to the fork accelerator TIMELESS when exposed to low levels of ROS. Elevated ROS levels disrupt PRDX2, whose dissociation from chromatin enforces the displacement of TIMELESS from the replisome slowing down fork progression [60]. When RAS overexpressing fibroblasts are maintained in culture for a long time, some cells restart to grow and can be isolated as colonies. The clones that have escaped senescence show an increased expression of the components of the fork protection complex, among them TIMELESS and Claspin. In these senescence-escaping clones, fork progression defects are suppressed in a TIMELESS-dependent manner [42].

#### 3.3.5. Global DNA Damage Signaling Induced Fork Slowing

Although speculative in the context of RAS-transformed cells, it is important to note that DNA damage, indirectly, through the activation of the DDR may actively impede fork progression. In non-cancerous cells, the absence of the replication checkpoint kinase CHK1 leads to massive DNA damage signaling, slowing down fork progression in an ATM-p53-dependent manner [61].

In human primary dermal fibroblasts exposed to hydroxyurea-induced RS, ATR slows down forks by targeting the MCM complex in a FANCD2-dependent manner [62]. When forks face discrete impediments such as those caused by interstrand crosslinks (ICLs), ATR signaling can also downregulate distant forks, although to a lesser extent than ICLs themselves [63]. When ATR signaling is activated in absence of damage by a light-induced oligomerization of TopBP1, replication forks slow in the bulk genome [64]. Altogether, these findings show that the ATR pathway restrains fork progression once activated (Figure 2D). In RAS-transformed cells, ATR [65] and ATM [31] signaling are activated, which may impact on fork progression. The fact that ATM is a major inducer of OIS [32] raises the question of whether ATM or ATR impact, at least partially, OIS through the control of fork speed.

## 4. Cytosolic DNA Sensing: A Link Between DNA Replication Stress and Oncogene-Induced Senescence

### 4.1. Oncogenic Stress and Cytosolic DNA Sensing by the cGAS-STING Pathway

The DNA sensor, cGAS, is critical in the process of either replicative- or DNA damage-induced senescence [33,35]. cGAS is similarly important to induce OIS upon H-RAS^v12^ overexpression [33,34,35,43]. The DNA sensor cGAS recognizes double stranded (ds)DNA species larger than 40 base-pairs (bp) producing cGAMP (cyclic guanosine monophosphate–adenosine monophosphate) as a second messenger and activator of STING (Stimulator of Interferon Genes) [66,67]. Senescence is associated with the secretion of cytokines and chemokines known as SASP. Overexpression of the main cytosolic 3’-exonuclease TREX1 decreases the level of SASP during OIS [68] and has been shown to block senescence [43].

It has been observed that oncogene expressing cells do suffer from mitotic failures [48] and accumulation of micronuclei [35,39]. As cGAS can relocate inside micronuclei, it has been proposed that they are the self-DNA promoting senescence and SASP [34,35]. Although the correlation between micronuclei accumulation and senescence has been consistently reported by several groups, it remains to determine whether micronuclei contain DNA in a form that potently activates cGAS. Indeed, it has been shown recently that nucleosomes block cGAS activity [69,70,71]. A recent article, using single-cell microscopy and fluorescent reporters of cGAS-STING signaling, strongly suggests that micronuclei are not potent activators of STING and Interferon (IFN) signaling [72]. Other cytosolic DNA species have been described at later stages of senescence. Fragments of single-stranded (ss)DNA of unknown sizes were visualized by immunofluorescence [73] and genomic double-stranded (ds)DNA were detected by PCR after sub-cellular fractionation [68]. RS leads to the accumulation of self-DNA in the cytosol (reviewed in [74]). This DNA is cleaved either at the level of stalled replication forks or DNA repair intermediates by the uncontrolled activity of different nucleases including the ds-DNA break repair nuclease MRE11 [66,75,76]. It remains to be determined whether the oncogenic RS can lead to the formation of cytosolic DNA from the unscheduled action of MRE11 nuclease. DNA break-induced senescence or OIS can both be suppressed by MRE11 inhibition [43,77]. Further characterization of cytosolic DNA upon oncogenic stress is therefore needed.

### 4.2. Is OIS a Real Thing?

One can ask whether senescence is detected in human samples, especially in early pre-cancerous lesions (as tumors would have escaped senescence to hyperproliferate and be immortal). Indeed, it has been a challenge to find markers of senescence in vivo and in patient samples, so that the presence of senescent cells that corroborate the OIS model of barrier to cancer formation are scarce (reviewed by [27,30]). Early studies have shown that DNA damage markers (e.g., γ-H2AX, markers of ATM/ATR activation) and senescence markers (HP1g SAHF) correlate in pre-cancerous (preneoplastic) lesions (colon adenoma versus carcinoma) [31,36]. Inhibition of ATM led to more tumors and more invasions [31].

The TRACERx consortium has recently shown that in various lung pre-invasive lesions, markers of senescent cells are readily detected, such as senescence-associated-β-galactosidase (SA-β-gal) staining or p21 positivity. It has been recently proposed that RS and chromosomal instability is driven by APOBEC3A activity in early pre-invasive lung lesions [78]. This APOBEC3A-mediated genomic instability may contribute to high cellular diversity in the early stage of cancer progression. Although senescent cells are detected, they are far less frequent than hyperproliferative cells (Ki67 positive) showing a heterogeneous cell population that oscillates between senescence/arrest of proliferation and hyperproliferation.

**Figure 3:**
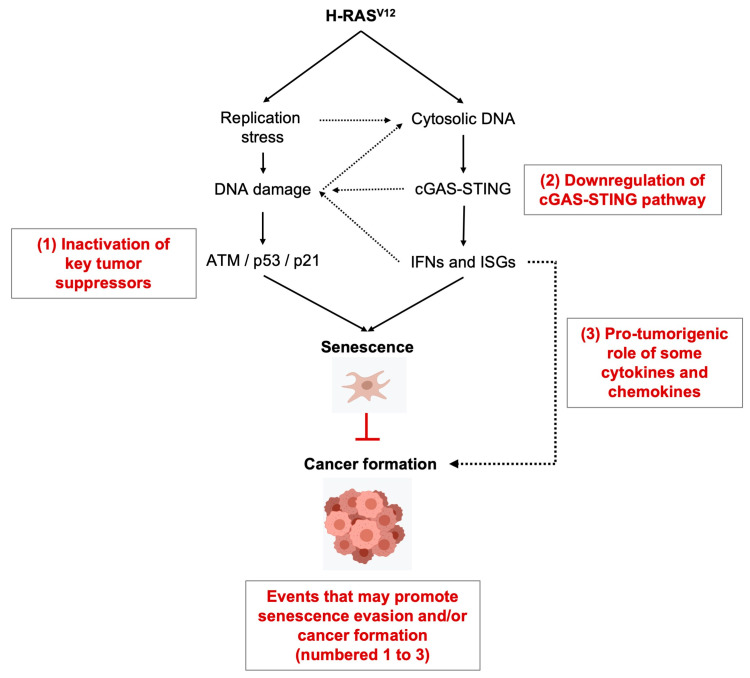
OIS is a barrier to tumorigenesis. Ras oncogene (such as H-RAS^v12^) can promote senescence by two main mechanisms: generation of RS and subsequent DNA damage signaling and activation of inflammatory response by the cGAS-STING pathway. Replication stress (RS) and cGAS-STING pathway are molecularly connected at different levels (shown with dashed arrows). Oncogenic stress may lead to the accumulation of cytosolic DNA because of genomic DNA damage and/or formation of micronuclei. Interferons (IFNs) and interferon stimulated genes (ISGs) have been proposed to generate RS and DNA lesions. Proliferation arrest upon senescence induction has been proposed to block tumorigenesis. As such, cancers do inactivate tumor suppressor genes, such as key factors of the DDR (1), or downregulate the cGAS-STING pathway (2). It has also been observed that some pro-inflammatory chemokines and cytokines can exert a pro-tumorigenic effect (3). Further details and references are in the main text.

If OIS is a real thing, and if the DDR is a barrier to cancer formation, one prediction is that the inactivation of DDR/checkpoints genes and senescence drivers in vivo in cancer samples would be selected. Indeed, in vitro it has been shown that inactivation of the DNA damage checkpoint, namely the ATM-CHK2-p53 axis, promotes first OIS escape and then proliferation and colony formation. If injected in immuno-deprived mice, these DDR inactivated RAS cells form more proliferative tumors [32]. In cancer samples it has been shown that, of course, TP53 is frequently inactivated, including lung cancers [3,15]. However, not only p53, but ATM mutation has been shown to correlate with RAS-mutated tumors in cancers [13,15]. Very interestingly, p21 (encoded by CDKN2A) is also associated with RAS signature [13,15]. In brief, most RAS-driven cancers are associated with the loss of the main inducers of senescence: namely p53, p21 or ATM (Figure 3) [31,32]. It is important to mention here that STK11/LBK1 (Serine/threonine kinase 11, also known as liver kinase B1) is also frequently mutated in K-RAS mutant lung adenocarcinomas [15]. STK11/LBK1 is a regulator of AMPK which impacts cell metabolism, its involvement in senescence or RS-response is not yet known. Recent works show that, notably, STING is epigenetically silenced in different types of cancers, including K-RAS/LKB1 mutated lung cancers [79,80]. Mitotic poisons that induce micronuclei were able to restore STING signaling and subsequently immune cells infiltration in the tumor environment. The cGAS-STING pathway is a senescence driver and a modulator of cancer cell rejection by the immune system; as such, cancer cells may benefit from inactivating this pathway (Figure 3).

## 5. Overcome Oncogenic RAS by Targeting Genome Instability in Cancer Cells: A Few Examples

**Figure 4:**
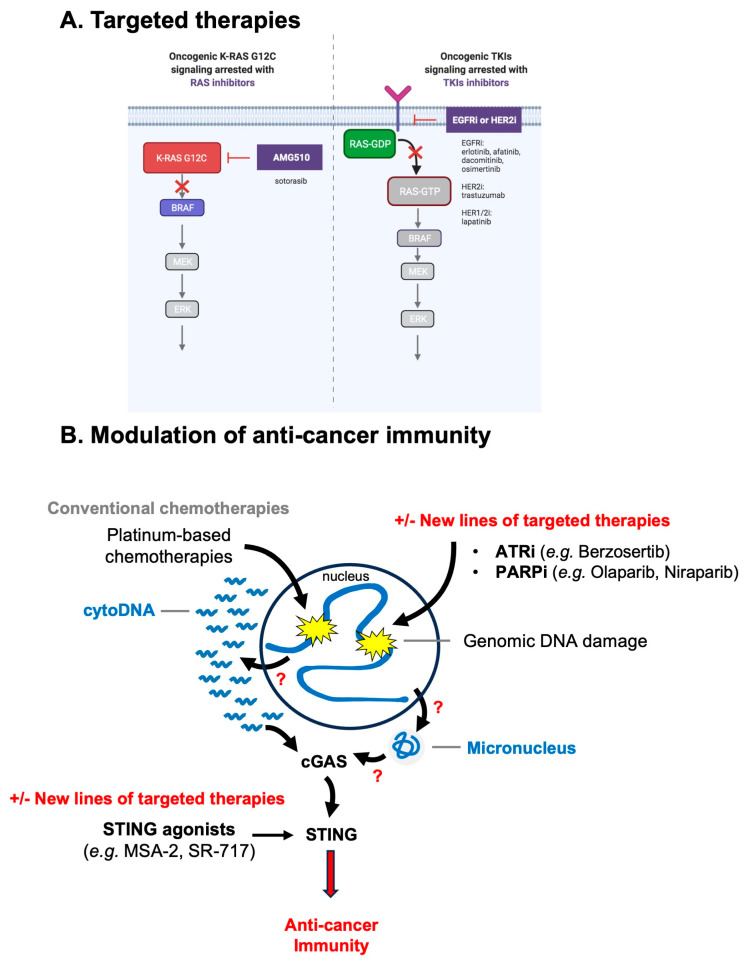
Targeted therapies, new lines of chemotherapies and cancer innate immunity. (**A**) Targeted therapies in the context of RAS-driven cancers, aim for instance at targeting directly the mutated RAS oncogene or the broad RAS signaling pathway. AMG510 binds to KRASG12C mutation. TKIs (tyrosine kinase inhibitors) such as EGFRi or HER2i are also under development to block RAS signaling. This figure has been created with Biorender.com. (**B**) Advances in new therapeutics are aimed at increasing the DNA replication stress and at promoting inflammatory signaling. Genomic instability and inflammation being connected, it has been reported that the benefit brought by replication stress-inducers (ATRi or PARPi) is mediated through the cGAS-STING pathway and anti-cancer immunity. Questions marks (in red) highlight here that the molecular mechanism that leads to cytosolic DNA and micronuclei formation upon such treatments, and which activates cGAS is not yet understood. STING agonists are new molecules that can promote STING signaling and subsequent anti-cancer immunity. These new lines of therapies show promising results in combination (indicated by +/-) with conventional chemotherapies (platinum-based DNA damaging agents) or with immune-checkpoint inhibitors (anti-PD1 or anti-PD-L1). See main text for details and references.

As cancer cells become ‘addicted’ to oncogenic signaling [14], it has been proposed that RAS oncogenic mutations can be directly targeted by small molecules. One of these compounds is AMG510 which targets the KRASG12C mutation (Figure 4A). AMG510 increases pro-inflammatory signals and has a good effect as single therapy or in combination with immune-checkpoint inhibitors [81]. In NSCLC patients, good regression of the tumors was observed even in patients that were not responding to prior intervention with classical chemotherapies (combination of Carboplatin, nivolumab, pemetrexed). In summary, AMG510 boosted the T cell-mediated anti-tumor response, improving the infiltration of T cells, notably of CD8+ T cells that were observed inside the tumors. The effect on T cell infiltration is concomitant with an increased IFN-γ upon treatment with AMG510 [81]. AMG510, under the commercial name Sotorasib, is now approved by the FDA for NSCLCs treatment. It is also possible to broadly inhibit RAS signaling by using tyrosine kinase inhibitors (TKIs), such as EGFR or HER2 targeting molecules (illustrated in Figure 4A). Despite these positive effects in pre-clinical models and clinical trials, little is known about the effect of RAS inhibitors or other RAS-targeted therapies on RS and senescence. RAS inhibitors might in fact impact broadly the functions of oncogenic RAS and affect the DNA replication process and senescence, having potentially positive and negative effects on the outcome of such therapies. Further work is needed to determine the impact of RAS-targeted therapies on genome stability and proliferation in cancer cells and pre-cancerous lesions, to further potentiate the benefit of such treatments.

As mentioned above, NSCLCs are somehow insensitive to classical chemotherapies that target genome stability. Using the RAS84 transcriptional signature, mentioned in the first section, it has been confirmed that cancers driven by oncogenic RAS activity are insensitive to current known compounds targeting DNA replication and mitosis [13]. It is thus fundamental to provide evidence for new small molecules to target lung cancers, as monotherapies or adjuvant therapies. In the next sections, we describe possible alternatives to current cisplatin usage, focusing on treatments that target RS (Figure 4B). Here, it is important to note that many RS inducers have toxic side effects that would eventually limit their usage.

### 5.1. Targeting the S-Phase Checkpoint: ATR Inhibition

SCLC tumors display a significantly higher expression of genes involved in cell cycle regulation, DNA damage signaling and DNA repair than NSCLC tumors [82]. In this study, the most differentially expressed gene is CHEK1. SCLCs are thus addicted to ATR and CHK1 for their survival. ATR and CHK1 inhibitors display a selective toxicity in SCLC cells. In opposition, NSCLCs are not sensitive to ATR-CHK1 inhibitors as monotherapies. SCLCs are very largely deficient for p53 and RB1 (retinoblastoma 1, master regulator of the cell-cycle), which may explain their sensitivity to ATR and CHK1 inhibitors. ATR depletion is synthetically lethal (more chromosomal breaks, subG1 cells) with oncogenic RAS-transformation at an early stage. However, the surviving cells deficient for ATR further transform and lead to more cancerous lesions in mice models [65] so that ATR targeting can confer both effects, a desirable effect on the elimination of cancerous cells and a negative effect that may promote cellular transformation and the emergence of resistant clones. Accordingly, the current usage of ATR pathway inhibitors is in combination with other treatments, especially DNA replication stress-inducers.

Targeting of ATR induces the regression of NSCLCs in mice models and human patients. Combination of Topoisomerase I inhibition (Topotecan) and ATRi (in this study Berzosertib) show good tumor response even in platinum-resistant patients. Combination exacerbates RS and DNA damages [83].

Low dose ATRi or CHK1i in monotherapies are well tolerated by cancer cells, but combination of both ATRi and CHK1i may go beyond the threshold of stress and of RS that cancer cells can bear [84]. The combination had good effects on survival and tumor regression in lung cancer xenograft models. However, we are skeptical about this strategy as CHK1i and ATRi to a lesser extent are both toxic to normal cells too. Indeed, in patients, one issue of targeting the ATR pathway is to find the right dosage and administration protocol to specifically kill cancer cells without impacting on healthy tissues.

### 5.2. Inhibition of PARP

Platinum-based drugs are the first-line therapy for lung cancer patients. However, platinum-based chemotherapeutics are poorly efficient as mentioned above. Precision medicine, Niraparib (PARP inhibitor, PARPi) and radiation have been shown to synergize to inhibit tumors both in vitro and in vivo in EGFR-mutated non-small cell lung cancer [85]. It is currently unclear why EGFR mutants are particularly sensitive to PARPi. Radiation plus niraparib have been shown to activate anti-tumor immunity, which appeared as increased CD8+ T lymphocytes and activated STING/IRF3 pathway [85] (see below for more details on immune modulation in cancer therapy).

Homologous recombination (HR) genes including ATM, BRCA1 and BRCA2 are frequently mutated in lung cancer patients. ATM-, BRCA1- and BRCA2-associated HR deficiencies represent approximately 14% of NSCLCs. NSCLCs HR-deficient cells are sensitive to PARP inhibitors like Olaparib [86]. HR deficiencies may be targeted through their sensitivity to acetaldehyde toxicity, as it has been shown in H1299 NSCLC carcinoma cell line depleted for BRCA2, and in other cancer cell lines from other types of cancers. The observed reduction in the viability of BRCA2-deficient H1299 NSCLC cells treated with aldehydes is like that of PARPi Olaparib [87]. Acetaldehyde is generated both endogenously, as a byproduct of cellular metabolism, and in response to exogenous factors, such as alcohol consumption. Acetaldehydes react with DNA, creating damage and crosslinks. It has been proposed that the compound disulfiram, an Aldehyde dehydrogenase inhibitor (ALDH1A1 and ALDH2), can sensitize HR-deficient cancerous cells [87].

Loss of ERCC1 (Excision repair cross-complementation group 1) is the most common DDR defect in NSCLCs, occurring in 30% to 50% of cases [88]. The median overall survival in NSCLC patients in the ERCC1-negative and ERCC1-positive patients was 11.8 months and 9.8 months, respectively. Suggesting that ERCC1 negative may be less aggressive or respond better to treatment [89]. Low ERCC1 expression is linked to cisplatin sensitivity in several types of cancer, including NSCLCs [88,90]. ERCC1 works within a heterodimer with XPF to cut DNA at the 5’ site of lesions. This process is thought to be the most important in processing platinum-induced DNA crosslinks. It has been further observed that PARP inhibition generates cytosolic DNA (in this case cytosolic chromatin fragments, CCFs, were observed) in an ERCC1-dependent manner in NSCLC cells [90,91]. PARPi-induced CCFs have been proposed to activate cGAS. PARPi induced the secretion of the chemotactic chemokine CCL5 in a cGAS- and STING-dependent manner and activated type I Interferon (IFN-I) signaling.

A new role of PARP-1 in controlling ROS levels upon EGFR TKI treatment has been discovered, with potentially broader implications for therapeutic targeting of the mechanisms that govern the survival of RAS oncogene-driven cancer cells. TKI resistance promotes PARP inhibitor sensitivity of EGFR mutant lung cancer cells [92]. The sensitivity of PARPi to TKIs has been demonstrated in vitro and in vivo and appears to be independent of any TKI resistance mechanism. Consequently, the efficacy of PARP inhibition must be evaluated in different models of TKI resistance.

### 5.3. Creating Breaks by Proton Irradiation

In KRAS mutant cells, fork progression slowdowns and increased levels of cytosolic dsDNA (with the DNA dye picogreen) are observed. In this setting, forks are very sensitive to proton irradiation [44]. KRAS mutant cells are not sensitized to CHK1i compared with WT cells [44]. CHK1i is toxic to a variety of cancer and normal cells, making it poorly targetable to RAS or KRAS mutants, as already mentioned above. These results suggest that proton-induced DNA damages can be used as a new trigger of DNA RS and combination with other drugs targeting replication or repair mechanisms need to be investigated.

PARPi (Olaparib) and RAD51 inhibitor (B02) radiosensitize cancer cell lines, i.e., lung and pancreatic, to proton and X-ray radiation [93]. Combination of these DNA repair inhibitors enhanced persistent DNA damage, delayed apoptosis, prolonged cell cycle arrest and senescence upon irradiation.

### 5.4. Combination of Replication Stress and Immunotherapy—Principle of DNA Damage-Induced Inflammation

Genomic instability has been demonstrated to correlate with the induction of inflammatory signaling [94]. A seminal work has reported that deficiency in the apical kinase of the DDR, ATM or the presence of DNA damage, such as upon exposing cells to IR and etoposide, resulted in the accumulation of cytosolic DNA molecules and the subsequent triggering of an IFN-I response [94]. Following articles have consistently shown that a large panel RS and DNA damage inducers promote the accumulation of cytosolic DNA, mainly through two distinct mechanisms: (i) the formation and subsequent rupture of micronuclei resulting from the missegregation of chromosomes during mitosis, and (ii) the aberrant processing of stalled replication forks [5,6,67,95].

The rationale of targeting cGAS-STING signaling and the production of cytosolic DNA to control inflammation stems from the observation that the response to chemotherapies in immuno-competent pre-clinical models depends on functional innate immunity. STING has been shown to be essential for promoting tumor rejection in immunocompetent mice treated with the anti-cancer drug topotecan [96].

As mentioned above, it has been shown that ERCC1-deficient NSCLC cells accumulate cytosolic DNA (here CCFs were observed) and subsequently induce IFN-I in response to PARP inhibition [91]. NSCLC cells treated with PARPi induce the cell surface expression of the PD-L1 immune-checkpoint inhibitor and produce a pro-inflammatory response, notably with the secretion of the CCL5 chemokine [91]. In patient tumor samples, ERCC1 deficiency is associated with increased levels of lymphocyte infiltration, suggesting that the IFN-I response observed in cultured cells also occurs in vivo and promotes the recruitment of immune cells. This was recently demonstrated in vivo in an elegant pre-clinical model of small cell lung cancer [97]. In these immunocompetent mice, anti-PD-L1, PARPi or CHK1i (inhibition of the CHK1 kinase of the S-phase checkpoint) have only a modest effect on tumor growth, if at all, when used alone. However, both PARPi and CHK1i have a strong effect on tumor growth and promote its regression when administered with anti-PD-L1 immunotherapy. This result was observed in mice proficient for the cGAS-STING pathway. In STING- or cGAS-depleted mice, the combination of PARPi/anti-PD-L1 or CHK1i/anti-PD-L1 had no impact on tumor growth [97]. These data show that under these circumstances, self-DNA sensing is essential to promote the immune rejection of cancer cells. Below, we describe further how STING signaling could be exploited in cancer therapies to enhance anti-cancer immunity, and notably whether these new types of treatments can be applied to NSCLCs.

### 5.5. Taking Advantage of Inflammation to Eliminate Cancer Cells: Development of STING Agonists

STING agonists are small molecules that activate STING and subsequent induction of interferon response artificially, in absence of biological activators (i.e., DNA). These categories of compounds will mimic IFN-I induction as observed after massive DNA damage or viral infection. The inflammation induced by these molecules may have the potential to attract and activate immune cells to boost anti-tumor immunity.

Recently, this strategy has proven to be very effective in xenograft models. One strategy is to enhance the immune response through activation of the inflammatory pathway. cGAS and STING are main mediators of cytosolic DNA sensing. cGAS is not a good target because it also impacts the DNA repair mechanism of homologous recombination (HR) and thus would have side effects [98,99]. As such, cGAS inactivation promotes DNA repair and promote cell survival to DNA damaging conditions [98,99]. Pan and colleagues have developed MSA-2, a small molecule agonist of STING which shows promising results on Lewis Lung (LL-2) carcinoma xenograft models. These LL-2 lung cancers are an interesting model as they are usually a model of ‘cold’ tumors, which are insensitive to immune-checkpoint inhibitors. MSA-2 shows a better survival of LL-2 xenografted mice and has an additive effect with PD-1 mouse monoclonal antibodies. PD-1 antibody alone had no effect [100]. This STING agonist is orally deliverable and shows an effect as a single agent or in combination with PD-1 or PD-L1 immune-checkpoint therapies [100] in lung and other cancer models. The combination may show this effect through the attraction and infiltration of immune cells, such as CD8+ lymphocytes. This can be summarized as turning ‘cold’ tumors into ‘hot’ ones. Another STING agonist, which is a non-nucleotide cGAMP mimetic, namely SR-717, shows promising effect on the B16.F10 Melanoma model and can reduce lung metastasis [101]. SR-717 has a good effect in combination with PD-1 or PD-L1 immunotherapies by promoting the infiltration of immune cells. If this line of treatment can sensitize NSCLCs to immunotherapy, is not yet known.

## 6. Conclusions

The combination of treatments that both increase DNA damage and foster the immune rejection of cancer cells might produce the best effects, in terms of tumor regression and to avoid the emergence of resistance to therapies. Our review focuses on recent progress made in non-small cell lung cancers. However, our discussion is broader in scope and can apply to other types of cancers as similar findings were recently obtained in ovarian, breast and colon cancers, to name a few. In these combination schemes, it will be important in the following years to implement diversification of the panel of available treatment. Then, it will be paramount to determine sequential treatments and maintenance treatments because of the multiclonal nature of cancers and of the rapid adaptation of cancer cells to new lines of treatments.

In order to improve anti-cancer therapies, the following unanswered questions remain to be addressed.

1. To develop robust markers of the cGAS-STING pathway to inform on which patient could benefit from an inflammation-based treatment, notably because cancers can silence or inactivate cGAS or STING.
2. To develop treatments and delivery methods that target specifically cancer cells. Replication-stress inducers and inflammation-based therapies show promising results but can impact both tumor and healthy cells.
3. To investigate how targeted therapies, and notably RAS inhibitors, affect the replication stress and senescence mediated by RAS mutations in different stages of cancers. The question here is whether we can better control the therapeutic benefit of RAS inhibitors.

## Data Availability

Not applicable.
